# Effect of Oral Infection of Mayaro Virus on Fitness Correlates and Expression of Immune Related Genes in *Aedes aegypti*

**DOI:** 10.3390/v12070719

**Published:** 2020-07-02

**Authors:** Barry W. Alto, Ayse Civana, Keenan Wiggins, Bradley Eastmond, Dongyoung Shin

**Affiliations:** Florida Medical Entomology Laboratory, University of Florida, 200 9th St. Vero Beach, FL 32962, USA; acivana@ufl.edu (A.C.); keenan.wiggins@gmail.com (K.W.); beastmond@ufl.edu (B.E.); dshin@ufl.edu (D.S.)

**Keywords:** mosquito, arbovirus infection, immune response, cost of infection, survival, reproduction

## Abstract

Mayaro virus is a mosquito-borne *Alphavirus* endemic to forests of tropical South America with a sylvatic cycle involving non-human primates and *Haemagogus* mosquitoes. Human infection with Mayaro virus causes a febrile illness and long-lasting arthralgia and cases are often associated with exposure to tropical forest habitats. Human movement between tropical forest habitats and urban settings may allow for imported cases and subsequent local transmission by domestic mosquito *Aedes aegypti*. The relative importance of *Ae. aegypti* as a vector of Mayaro virus may depend on the pathogenic effects of the virus on fitness correlates, especially those entomological parameters that relate to vectorial capacity. We performed mosquito infection studies and compared adult survival and fecundity of females from Brazilian and Floridian populations of *Ae. aegypti* following oral ingestion of uninfectious (control) and Mayaro virus infectious blood. Mayaro virus infected and refractory mosquitoes had similar or 30–50% lower fecundity than control (unexposed) mosquitoes, suggesting a reproductive cost to mounting an immune response or phenotypic expression of refractoriness. Survival of adult female mosquitoes and targeted gene expression in the Toll and IMD pathways were not altered by Mayaro virus infection. Adult lifespan and fecundity estimates were independent of measured viral titer in the bodies of mosquitoes. The lack of adverse effects of infection status on female survival suggests that Mayaro virus will not alter vectorial capacity mediated by changes in this parameter.

## 1. Introduction

Mayaro virus (MAYV) was first isolated in 1954 from Trinidad [1]. Mayaro virus is an *Alphavirus*, family Togaviridae in the Semliki Forest Antigenic Complex [2] and is classified into three genotypes (D, L, and N). Genotype D includes strains from Peru, Bolivia, Venezuela, Trinidad and Tobago, and French Guiana. Genotype L comes from Haiti and Brazil [3] and genotype N comes from Peru [4]. Mayaro virus causes a self-limiting febrile illness characterized by headache, rash, nausea, musculoskeletal pain, and photophobia [2]. However, severe and debilitating arthralgia may persist for months in some cases, raising public health concerns [2,5]. Increased international travel and spread of potential mosquito vectors has contributed to enhanced risk of local transmission of mosquito-borne arboviruses in new regions, including most recently Zika, chikungunya, and dengue viruses. Mayaro virus, an *Alphavirus* related to chikungunya, has caused recent epidemics in the Brazilian states of Pará in 2008 [6], Mato Grosso in 2012 [7], Goiás in 2014–2016 [8] and in Bolivia [9]. Detection of MAYV or antibodies in potential vertebrate reservoirs has also been observed outside of known established regions, including Haiti in 2015 [3], and parts of Europe (Netherlands in 2008) [10], suggesting the potential for geographic expansion.

The sylvatic (enzootic) cycle of MAYV primarily occurs between *Haemagogus* mosquitoes and non-human primates [9,11]. *Haemagogus janthinomys* is considered the primary vector [9,11,12,13]. Human infections are associated with exposure to forest environments, which likely represent spillover from the sylvatic cycle by *Haemagogus* mosquitoes [14,15]. Mayaro virus belongs to the Semliki Forest Complex, as do newly emergent chikungunya virus lineages, suggesting it may have the potential to become urbanized and a public health concern [15]. Additionally, the occurrence of MAYV cases is situated near neotropical cities with *Aedes aegypti*, thus placing MAYV and potential urban vectors near one another [2]. Experimental studies to determine vector competence of domestic mosquitoes have demonstrated susceptibility to infection and potential transmission by *Ae. aegypti* [16,17] and *Ae. albopictus* [17,18], both known vectors of emergent arboviruses including Zika, chikungunya, dengue, and yellow fever. The intensity of MAYV transmission by *Ae. aegypti* is partially determined by entomological parameters such as biting rate, daily survival, vector competence, extrinsic incubation period, and mosquito population density. However, susceptibility to infection and refractoriness to MAYV (i.e., a phenotype refractory to infection) may incur fitness costs in *Ae. aegypti* and alter select entomological parameters. However, most mathematical models characterizing risk of arbovirus transmission assume negligible pathogen-induced changes in these entomological parameters.

Arbovirus infections have the potential to cause cytopathic effects in mosquitoes, including anatomical features associated with barriers to virus transmission such as the midgut [19,20] and salivary gland tissues [21,22]. Ingestion of arbovirus-infected blood elicits an immune response in mosquitoes [23,24,25] which may be associated with metabolic costs and associated trade-offs between immunity and fitness correlates. Further, exposure without arbovirus infection (e.g., a refractory phenotype) [26,27] or progression of status of an infection (non-disseminated infection and disseminated infection) may have unanticipated consequences for altered mosquito life history traits, some of which may contribute to their ability to transmit arboviruses (e.g., longevity, blood feeding behavior, reproduction). Consistent with this phenomenon, studies have shown that arbovirus infection has adverse effects on mosquito fecundity, survival, and blood-feeding behavior [27,28,29,30,31]. However, other studies have shown no observable effects [32] and even beneficial effects on mosquito fitness correlates [33,34]. Further, the magnitude in which arboviruses alter mosquito biology depends on taxonomic relationships of viruses and mosquitoes as well experimental conditions and mode of infection (horizontal versus vertical transmission) [32].

The goal of this study was to determine whether oral infection of MAYV causes decreased survival and fecundity in female *Ae. aegypti*, a potential urban vector of MAYV. We conducted laboratory infection studies using local geographic populations of *Ae. aegypti* from Brazil and United States. Brazil was chosen as an MAYV endemic location with high potential for epidemics and the potential to establish an endemic cycle [2]. Florida in the USA was chosen as a region associated with high numbers of imported cases and recent local transmission of emerging arboviruses, such as Zika, chikungunya, and dengue, thus suggesting other arboviruses may emerge that may utilize *Ae. aegypti* as a potential vector. We observed that mosquitoes that ingested MAYV infectious blood had lower fecundity than those individuals that fed on uninfectious blood (controls), especially those mosquitoes refractory to infection. Survival of mosquitoes that ingested infectious MAYV blood did not differ from those individuals that ingested uninfectious blood (controls).

## 2. Materials and Methods

### 2.1. Mosquito Populations and Rearing

Two populations of *Ae. aegypti* used in these laboratory studies were from field collections of immature stages from water-holding artificial containers in Stuart, Florida, United States and Rio de Janeiro (Urca), Brazil. The F_2_ and F_3_ progeny of the Florida and Brazil populations of *Ae. aegypti*, respectively, were used in all experiments. To synchronize hatching, mosquito eggs were hatched in 1 L of deoxygenated water prepared using an insulated vacuum container powered with an electronic pump. Within one hour after hatching, mosquito larvae were transferred to plastic rearing pans (50 cm in length, 40 cm in width, 7.6 cm in height) along with tap water and 2.7 g of larval food consisting of equal parts liver powder and brewer’s yeast. A density of approximately 200 larvae per liter of water was maintained during rearing larvae. When pupae developed, they were transferred to water-filled cups and placed in Bugdorm insect rearing cages (30 cm^3^) and held at 28 °C and a 12 h light and 12 h dark photoregime in a climate-controlled room. Adults were held in cages for five days to allow for mating and provided with 10% sucrose solution and water from cotton wicks. One day before providing *Ae. aegypti* blood containing MAYV, mosquitoes were anesthetized with carbon dioxide and transferred to cages with mesh screening (10 cm in height, 10 cm in diameter, 50 females/cage) with access only to water.

### 2.2. Mayaro Virus Isolate and Propagation

An isolate of a prototype strain of MAYV (TRVL 4675, GenBank: MK070492.1) was provided by the USA Centers for Disease Control and Prevention. The virus was originally isolated from an infected human in Trinidad in 1954 and passaged in primary cultures (hamster kidney, chick embryo, mouse embryo) and cell lines (BHK-21 and Vero cells). We propagated the virus three times in tissue culture consisting of monolayers of African green monkey kidney (Vero) cells (American Type Culture Collection, No. CCL-81, Manassas, VA, USA) and media (GIBCO^®^, Grand Island, NY, USA; Media 199, 10% fetal bovine serum, 2% penicillin–streptomycin). Propagation of MAYV was performed in an incubator at 37 °C and 5% carbon dioxide atmosphere. Following 48 h of incubation and associated monolayer destruction, media were collected by aspiration and combined with defibrinated bovine blood (Hemostat, Dixon, CA, USA) as the MAYV-infected blood to be fed to mosquitoes (3:1 ratio of blood to media suspension). MAYV-induced cytopathology caused detachment of part of the monolayer and so collection of media also included cells. Similar methods were used to create control blood meals (no virus), except that cell cultures were inoculated with media only.

### 2.3. Infection Study

Female mosquitoes aged eight days were allowed to feed on MAYV infectious blood or uninfectious blood (control) using an artificial membrane feeding system (Hemotek, Lancashire, United Kingdom) with hog intestine membranes. Adenosine triphosphate (ATP) at 0.005 M was added as a phagostimulant to the blood meals. Samples of blood were taken from the virus–blood suspension at the time of feeding to gauge the concentration of MAYV ingested by the adult mosquitoes. The titer of infectious blood meals was 7.0 log_10_ plaque forming units (pfu)/mL. The viral titer of MAYV-infected blood ingested by mosquitoes is higher than typical viremias in primates (rhesus monkeys, 5.7–5.9 log_10_ pfu/mL; humans, 5.0 log_10_ pfu/mL) [35,36]. However, the use of a higher titer allowed us to achieve sufficient numbers of mosquitoes in various states of infection following exposure (refractory, non-disseminated infection, disseminated infection) and to assess both midgut infection and escape barriers. Immediately following feeding trials, mosquitoes were anesthetized and sorted using light microscopy (10×). Fully engorged females were transferred back to their original cages and provided with water and 10% sucrose pads and an oviposition substrate. Females could lay eggs and five days following the infectious blood meal they were allowed to feed on uninfectious blood using similar methods for second oviposition. Eggs from the second gonotrophic cycle only were examined because the extrinsic incubation period may exceed the length of time for the first gonotrophic cycle [37,38]. We reasoned that our ability to observed costs of infection may be more likely in an advanced state of infection. Fully engorged mosquitoes were transferred individually to 37 mL plastic tube cages (8 × 3 cm, height × diameter) along with an oviposition cup and water and 10% sucrose pads. Each tube held a single mosquito and was fitted with a removable screen lid. Mosquitoes were checked daily and mortality was recorded. Dead adults were immediately collected and stored at −80 °C for later processing. Eggs were stored in the same incubator at 28 °C, approximately 60–80% humidity, and at a 14:10 h light:dark photoperiod for at least one week and enumerated using light microscopy at 10× magnification.

Mosquitoes were tested for infection on the day they died following ingestion of MAYV-infected blood. Parental mosquitoes were dissected using sterile forceps with the aid of light microscopy (10×). Bodies and legs of individual mosquitoes were tested separately as indicators of susceptibility to MAYV infection and disseminated infection [17]. Bodies and leg samples from each female were placed in separate tubes with 1 mL media (Media 199, GIBCO^®^, Grand Island, NY, USA) and triturated mechanically in a TissueLyser (Qiagen, Inc., Valencia, CA, USA) with two steel bearings (4.4 mm in diameter) and clarified by centrifugation.

### 2.4. Viral RNA Isolation and qRT-PCR

Viral RNA was extracted from 140 µL of mosquito body and leg homogenates using the QIAamp Viral RNA Mini Kit (Qiagen, Valencia, CA, USA) and eluted in 50 µL of buffer according to the manufacturer’s protocol. MAYV RNA was identified using the Superscript III One-Step qRT-PCR with Platinum^®^ Taq kit by Invitrogen (Invitrogen, Carlsbad, CA, USA). Primers had the following sequences: forward, 5′-TGGACCTTTGGCTCTTCTTATC-3′: and reverse, 5′-GACGCTCACTGCGACTAAA-3′. The probe sequence was: 5′-/56-FAM/TACTTTCCTGCTGCAAGGGCTCTT/3BHQ_1/-3′ (Integrated DNA Technologies, Coralville, IA, USA). Primers were designed to target a nonfunctional structural polyprotein precursor gene (GenBank accession DQ4873691). Quantitative RT-PCR was performed with the CFX96 Real-Time PCR Detection System (Bio-Rad Laboratories, Hercules, CA, USA). The program for qRT-PCR was as follows: 50 °C for 30 min, 94 °C for 2 min, 39 cycles at 94 °C for 10 s and 60 °C for 1 min, and 50 °C for 30 s. The amplicon formed by this assay is 91 base pairs long. The titer of MAYV in mosquito samples was determined using a standard curve method by comparing cDNA synthesis for serial dilutions of MAYV together with plaque assays on serial dilutions of MAYV, expressed as plaque forming unit equivalents (pfue)/mL [39].

### 2.5. Statistical Analyses

Contingency table analysis was used to compare susceptibility to infection and disseminated infection between geographic populations of *Ae. aegypti* from Brazil and United States. Treatment effects on adult survival were compared using a regression analysis of survival data based on the Cox proportional hazards model (PROC PHREG, SAS 9.22), testing for effects of geographic origin of mosquito (Brazil, USA), infection status (control, infected, refractory) and the origin by infection status interaction using Type 3 Tests (*Wald Chi Square*). Infection status could be further distinguished between individuals with disseminated and non-disseminated infections. However, disseminated infection co-varies with time and thus adult survival. Therefore, infected individuals include both individuals with disseminated and non-disseminated infection. Analysis of variance was used to determine treatment effects on number of eggs laid. To determine whether viral load influenced adult survival and the number of eggs laid, we performed separate regression analyses for each mosquito population with viral titer and adult survival and viral titer and number of eggs laid only for mosquitoes with disseminated infections.

### 2.6. Gene Expression

Following similar methods for the MAYV infection study, mosquitoes were allowed to feed on MAYV-infected bovine blood or control (uninfected) blood using the Hemotek feeding system. Cohorts of mosquitoes were samples at 0, 4, 8, 12, 24, 72, and 168 h post-feeding and stored at −80 °C for later processing. Total RNA from 10 mosquito bodies was extracted for each biological replicate using Trizol and primers specific to the genes of interest that involved in mosquito immune pathway to *Alphaviruses* (Table 1) were designed to determine the targeted gene expression by quantitative Real-Time PCR (qRT-PCR) [40]. The targeted genes have key roles in Toll and IMD pathways and these pathways have shown effects on infection of *Alphaviruses*, especially O’nyong’nyong and Sindbis [41,42,43,44]. The related gene expression level was normalized to the expression of the *Ae. aegypti* ribosomal protein S7 gene (GenBank Accession # AY380336) [45]. The levels of gene expression were compared to non-infectious blood-fed mosquitoes. The gene expression in each group was compared by delta-delta Ct value analysis. Standard deviation was calculated. The gene expression difference between before and after MAYV infection was determined by Kruskal Wallis nonparametric analysis. The same samples for the gene expression study were titrated for MAYV using qRT-PCR with MAYV specific primer. The standard curves were generated by serial dilution of MAYV stock. The titers of each sample were determined with the obtained standard curve.

## 3. Results

There were significant effects of geographic population on susceptibility to infection, with higher rates of infection observed in *Ae. aegypti* from Brazil than Florida (*χ*^2^ = 16.65, *df* = 1, *P* < 0.0001, Table 2). Similarly, there were significantly higher rates of disseminated infection observed in *Ae. aegypti* from Brazil than Florida (*χ*^2^ = 7.39, *df* = 1, *P* = 0.0066, Table 2). There were no significant differences in the body viral titer between *Ae. aegypti* from Brazil and Florida for individuals with non-disseminated infections (*F* = 0.24, *df* = 1.45, *P* = 0.6251, Table 1) and disseminated infections (*F* = 1.03, *df* = 1.70, *P* = 0.3146, Table 2). Similarly, there were no significant differences in the viral titer of leg samples between geographic populations of *Ae. aegypti* from Brazil and Florida (*F* = 2.57, *df* = 1.71, *P* = 0.1136, Table 2).

**Figure 1:**
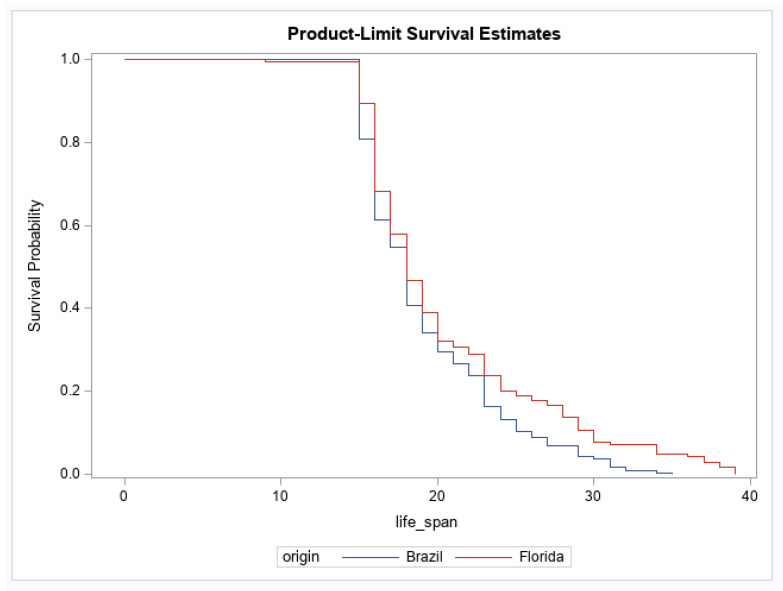
Female survival for *Aedes aegypti* originating from Brazil and Florida. Survival probabilities combine mosquitoes fed uninfected (control) and Mayaro virus-infected blood (Brazil, *n* = 214, Florida, *n* = 180).

**Figure 2:**
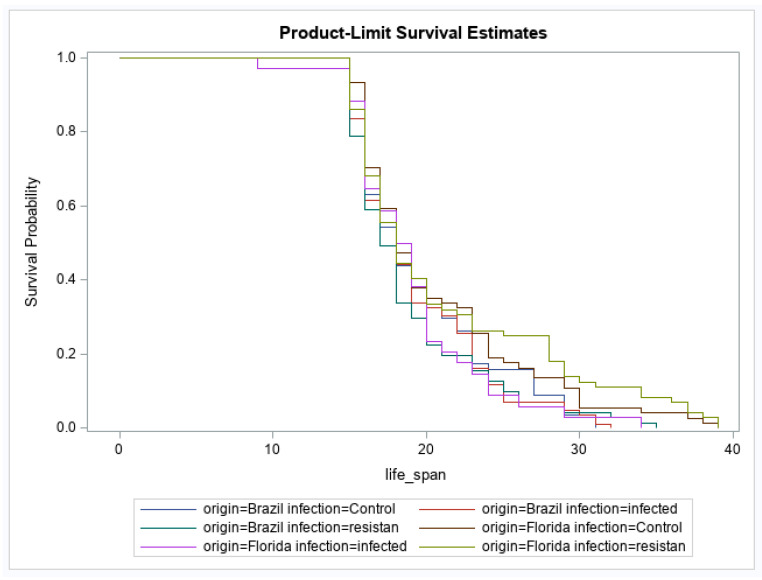
Effects of Mayaro virus (MAYV) infection on adult female survival in *Aedes aegypti*. Survival probabilities are illustrated according to geographic origin of *Ae. aegypti* and infection status (Brazil control, *n* = 57; Brazil infected, *n* = 86, Brazil resistant/refractory, *n* = 71; Florida control, *n* = 74; Florida infected, *n* = 34, Florida resistant/refractory, *n* = 74).

For adult survival, there were significant effects of geographic origin and origin by infection status interaction (Table 3). The main effect of infection status was not significant (Table 3). The origin effect showed that the geographic population of *Ae. aegypti* from Brazil had a higher probability of reduced survival over time than *Ae. aegypti* originating from Florida (Figure 1). For the interaction, after correcting for multiple comparisons, no groups were significantly different from one another (Figure 2). No other pairwise comparisons were significantly different from one another.

**Figure 3:**
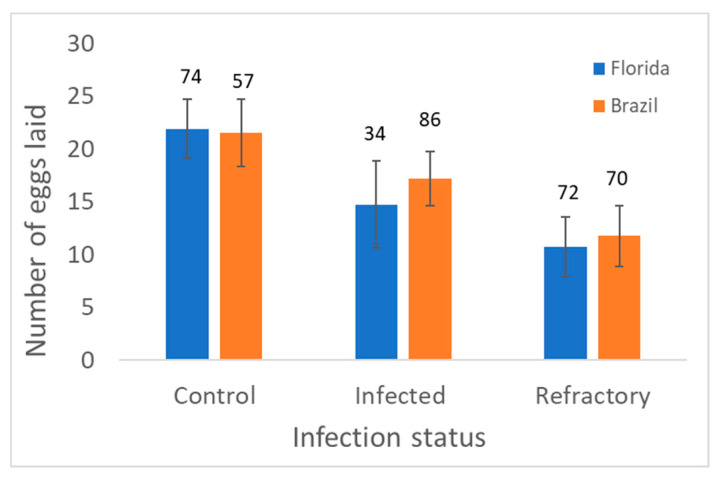
Least squares mean and standard error for the number of eggs laid by adult females in Control (unexposed) and MAYV-infected treatments (exposed) by infection status (infected or resistant/refractory) in *Aedes aegypti*. Numbers above bars show the number of female mosquitoes measured.

Analysis of variance of number of eggs laid (approximate fecundity following exposure to MAYV) showed a significant effect of infection status, but no significant effects of geographic origin and interaction (Table 4). Averaging over geographic populations, refractory and infected mosquitoes had lower fecundity than control individuals (Figure 3). However, only refractory mosquitoes had significantly lower fecundity than control mosquitoes. Fecundity was highest for mosquitoes in the controls (Figure 3).

**Figure 4:**
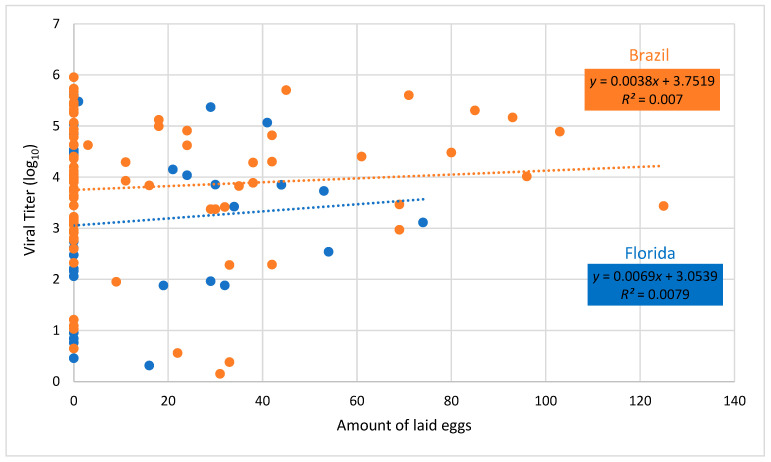
Body viral titer and number of eggs laid of female *Aedes aegypti* from Brazil and Florida infected with Mayaro virus. Dashed lines drawn through viral titer values show the best fit for Brazil and Florida.

**Figure 5:**
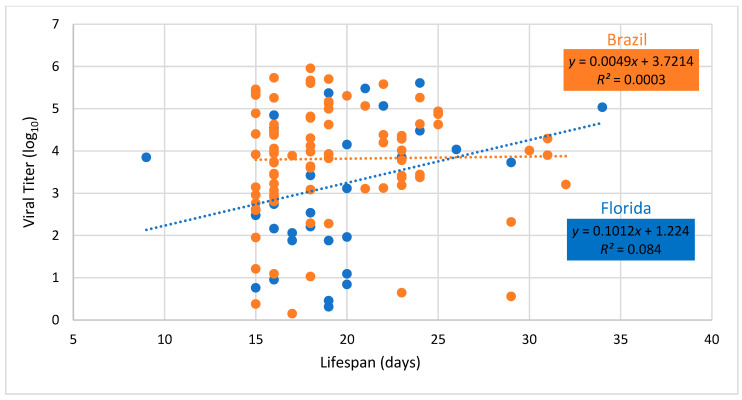
Body viral titer and lifespan of female *Aedes aegypti* from Brazil and Florida infected with Mayaro virus. Dashed lines drawn through viral titer values show the best fit for Brazil and Florida. Adult lifespan is measured from date of eclosion to death.

Regression of fecundity versus body viral titer showed no significant relationship for either Brazilian or Floridian populations of *Ae. aegypti* (Brazil, *F* = 0.59, *df* = 1.84, *P* = 0.4446; Florida, *F* = 0.26, *df* = 1.32, *P* = 0.6164, Figure 4). Similarly, regression of lifespan versus body viral titer showed no significant relationship for either Brazilian or Floridian populations of *Ae. aegypti* (Brazil, *F* = 0.02, *df* = 1.84, *P*=0.8808; Florida, *F* = 2.93, *df* = 1.32, *P* = 0.0965, Figure 5).

The titer of MAYV and level of expressions in the selected genes did not show a significant difference between the populations of mosquitoes or MAYV infection status across the sampling time point (0, 4, 8, 12, 24, 72, and 168 h) (*P* > 0.05, Table 5). Using Mann Whitney testing, we determined the difference of titer between populations in each time point and time point within populations (*P* = 0.8048, Mann–Whitney *U* = 22). Although the result was not statistically supported, the Brazil population showed higher titer at 72 h compared to Florida population of mosquitoes (Table 6). The samples from Florida population had a large deviation among the biological replications, but this titer difference in the data set was notable. We determined gene expression level difference between population over time and each time point within population using Kruskal–Wallis non-parametric comparison analysis. Although the gene expression results did not show a statistical difference, Caspar and SPZ genes in the Brazil population increased gene expression level at a 168-h time point (*P* > 0.05, Kruskal Wallis statistic = 2.591 for Caspar, and Kruskal Wallis statistic = 4.875 for SPZ), while Myd88 gene in the Brazil population showed decreased gene expression at the same time point (*P* > 0.05, Kruskal Wallis statistic = 2.033). The increased gene expression in Caspar was higher in the non-infectious blood-fed group, but they were still not supported by statistical analysis. The infected Florida population showed increased gene expression of rel2 at the 168-h time point, but it was not statistically significant (*P* > 0.05, Kruskal Wallis statistic = 2.05).

## 4. Discussion

Mayaro virus is an emerging mosquito-borne arbovirus in the Americas, which has the potential to enter an urban cycle involving humans and domestic mosquitoes, most likely *Ae. aegypti*. The propensity of *Ae. aegypti* to serve as a potential vector depends, in part, on the influence MAYV has on life history attributes of *Ae. aegypti*. Life history traits that contribute to parameters of vectorial capacity (longevity, feeding rates, reproduction) may alter the relative importance of *Ae. aegypti* as a vector. To address this topic, we challenged two geographic populations of *Ae. aegypti* with oral infection with MAYV, along with controls (uninfectious blood), and measured fitness correlates, including number of eggs laid (a proxy for fecundity) and adult survival.

We observed that the geographic populations of *Ae. aegypti* responded differently with infection to MAYV. Here we show that the Brazilian population of *Ae. aegypti* is 40% more susceptible to MAYV infection and exhibits 40% higher disseminated infection than the Florida population of *Ae. aegypti*. These findings differ from infection studies with chikungunya virus (CHIKV), comparing infection between *Ae. aegypti* from the USA and the Neotropics which demonstrated that transmission potential (saliva infection), and not disseminated infection of CHIKV significantly differed between geographic populations of *Ae. aegypti* [46]. In contrast, another study showed no significant differences between USA (Key West and Okeechobee, FL) and Brazil (Macapá and Rio de Janeiro, Brazil) for disseminated infection of chikungunya virus. Differences in observed results may be associated with virus-specific responses and genetic variation among geographic populations of mosquitoes [47]. For example, the geographic population of *Ae. aegypti* from Brazil had lower survival rates compared to the Florida populations. Thus, higher infection and disseminated infection rates of *Ae. aegypti* from Brazil than Florida is countered, in part, by lower survival rates. Additionally, MAYV has been circulating in the Americas for far longer than CHIKV and may simply have different vector competence barriers.

Geographic differences in infection and disseminated infection observed in *Ae. aegypti* are consistent with other studies showing variation in vector competence among geographic strains of *Ae. aegypti* for infection with *Alphaviruses* and *Flaviviruses* (chikungunya, yellow fever, dengue) [46,48,49,50,51,52]. Previous studies have documented a wide range of variation in vector competence to transmit dengue viruses among populations of *Ae. aegypti*, even within Brazil [53]. Along the same lines, similar studies have shown variation in vector competence of geographic populations of other potential vector species, *Ae. albopictus*, for dengue-1 virus [54]. Taken together, these studies suggest a wide range of phenotypic responses in infection and transmission potential among mosquito vectors, presumably attributable to the mosquito genotype. Additionally, recent studies have demonstrated that variation in vector competence and associated dengue viral load is associated with specific genes that underpin antiviral responses [55]. In the current study, viral titer in mosquito tissues did not vary between the two geographic populations of *Ae. aegypti* at the time of testing, suggesting that viral replication per se was not the mechanism responsible for the observed differences in susceptibility to infection and disseminated infection. However, viral titer was only measured in mosquito tissues at a single end point, and so it is unclear whether temporal differences in MAYV viral titer occur during infection. Additionally, viral titer was not measured within the midgut early after infection, which may be another determinant for rates of viral dissemination.

The gene expression portion of the study, along with titer in different populations of *Ae. aegypti* mosquito did not show significant differences between populations and time after infection. However, there are some notable changes in titer at 72 h and in some genes including Myd88, Caspar, and rel2 at 168 h after infection. This result was not statistically significant because of the large standard deviation among biological replicates. In addition, it is possible that our sampling did not capture time points critical to the expression of these genes. Samples collected in different time points in a range of 72 to 168 h during infection may reveal differences. Moreover, this study used the whole body for gene expression study, and so tissue-specific gene expression may have been hindered. Further studies including samples collected at various time points between 72 and 168 h and various tissue such as midgut or salivary gland tissue may be able to reveal the vector competence responsible for gene pathways. Gene expression and titer studies can be supported with titers and gene expression studies at slightly different time points as shown in this study. We can suggest that Brazilian population efficiently responded to titer change in immune response pathways and altered their vector competence. Overall, we did not find evidence to suggest that infection altered survival distributions of *Ae. aegypti* following oral exposure with MAYV, suggesting negligible effects of MAYV infection on survival in *Ae. aegypti* females. Our observation contrasts with other *Alphavirus* infection studies that show a reduction in survival of adult mosquitoes such as *Culiseta melanura* and *Culex tarsalis* infected with Eastern equine encephalitis and Western equine encephalitis, respectively [29,56]. Similarly, for *Flaviviruses*, other studies that have shown higher survival in West Nile virus (WNV) susceptible and unexposed *Culex pipiens* mosquitoes than refractory mosquitoes [26]. Along the same lines, a study of a field population of *Ae. aegypti* from Rio de Janeiro, Brazil orally challenged with dengue-2 virus showed that unexposed (control) mosquitoes lived longer than infected individuals, but infected individuals lived even longer than those exposed but negative (refractory) individuals [27]. Additionally, Zika infection decreased locomotor activity in a population of *Ae. aegypti* from French Polynesia [57]and reduced lifespan of populations of *Ae. aegypti* from Brazil [58,59]. In contrast, a large-scale study showed similar survival among cohorts of *Ae. aegypti* having previously fed on dengue serotypes 1-4 infected patients in Vietnam, regardless of whether mosquitoes exhibited infection or not, including uninfected controls [60]. The previous study measured survival during a 12-day observation period, and it is unclear whether dengue virus influences survival later in life. These latter studies are consistent with a meta-analysis showing negative effect on fitness for horizontally transmitted arboviruses [32]. However, arbovirus-induced changes in mosquito life history traits are likely to have nuanced effects depending on viral genotype, mosquito genotype and environmental factors. For example, West Nile virus (WNV) induced changes in survival of *Culex pipiens* dependent on geographic origin and environmental temperature [61]. Specifically, oral exposure to WNV decreased survival among *Cx. pipiens* from Gainesville, FL relative to unexposed individuals at 31 °C. In contrast, exposure to WNV enhanced survival among *Cx. pipiens* from Vero Beach, FL relative to unexposed individuals at 27 °C. These results demonstrate that arbovirus exposure may decrease or increase fitness correlates depending on other factors [61]. Furthermore, a history of passaging in mostly mammalian cells may compromise virus fitness in mosquitoes and associated mosquito life history traits [62,63]. Additionally, other studies have demonstrated that the mode of transmission (horizontal versus vertical) may influence the impact of arboviruses on mosquito fitness [32]. Arboviruses vertically transmitted may select for lower virulence. For example, vertical transmission of LaCrosse virus (LACV) is an overwintering mechanism allowing mosquito mothers to act as reservoir vectors and transmit the virus to their offspring following temperate winters [64,65]. Under this circumstance, theory and empirical data suggests that parasites should select for decreased virulence [66,67]. Consequently, previous studies have shown neutral, beneficial, and deleterious effects of LACV on vector *Aedes triseriatus* [33,68,69]. In the current study, the lack of observed negative effects of MAYV exposure on *Ae. aegypti* suggest negligible effects of infection or that mosquitoes were able to compensate for possible negative effects, perhaps through regular access to nutrition (sucrose) as adults, or alterations in other life history traits. In addition, no difference in the immune-related gene expression levels after the MAYV infection and titers have been observed in different time points. Although there is the possibility that immune signaling pathways can be involved in the defense mechanism towards MAYV infection, those selected genes have key roles in each of the immune signaling pathways such that Toll and IMD pathways typically respond to other *Alphaviruses* [41,42,43,44,70]. These results support the notion that MAYV may not elicit a strong immune response in *Ae. aegypti*. Regardless, the lack of adverse effects of MAYV on female survival in *Ae. aegypti* suggests that this parameter of vectorial capacity will not be limited by MAYV.

Although MAYV exposure did not strongly influence adult survival, we observed negative effects on fecundity. Specifically, we observed a fitness cost of a 50% reduction in number of eggs laid associated with refractory phenotypes of MAYV infection. Similar findings have been shown for *Ae. aegypti* following ingestion of blood infected with the ECSA lineage of chikungunya virus [71]. Specifically, chikungunya virus-infected mosquitoes laid fewer eggs and exhibited down regulation of six transcripts in the egg laying pathway than mosquitoes fed uninfected blood, thus linking *Alphavirus* infection to alterations in the expression of genes involved in the reproductive cycle. Consistent with these findings, other *Alphaviruses* have been shown to inflict a cost on fecundity (Eastern equine encephalitis virus and *Culiseta melanura*; Western equine encephalitis virus and *Culex tarsalis*) [29,56]. For *Flaviviruses*, similar findings have been shown for dengue-2-infected *Ae. aegypti* [27] and WNV-infected *Cx. tarsalis* [31], but not for WNV-infected *Cx. pipiens* [26] and Zika-infected *Ae. aegypti* [60,61]. In contrast, Zika infection decreased the number of eggs produced compared to an uninfected population of *Ae. aegypti* from Brazil [62]. In the current study, we observed that fecundity was impaired in mosquitoes with refractory phenotypes, perhaps attributable to trade-offs in energy expenditures between reproduction and immunity. Mounting an immune response in response to ingestion of pathogen-infected blood is metabolically costly (e.g., *Plasmodium*-induced reduced fecundity in *Anopheles*; dengue-induced transcriptional responses in *Ae. aegypti*) [72,73,74] and therefore should reduce mosquito fitness [23,24]. Individuals that are refractory to infection may suffer even greater fitness costs than individuals that are susceptible to infection [26,75]. However, what is not clear is whether these individuals mount a greater immune response or differ in other ways, making them less likely to become infected.

Arbovirus infection and replication may be costly to the mosquito. This assumption predicts that individuals with higher viral loads should incur greater negative fitness-associated costs. However, we did not observe relationships between viral body titer and adult survival or fecundity, suggesting low virulence of MAYV on the mosquito vector *Ae. aegypti*. However, we cannot rule out the possibility that viral load may be associated with other costs that were not measured in this study (e.g., nutrient reserves; lifetime fecundity; avidity to blood feed) [58,61,76] or under more stressful environmental conditions.

## Figures and Tables

**Table 1:** Primer sequences for testing immune response related gene expressions.

Gene ID	Description	Primer Sequences
AAEL007696	Rel1A	Forward CTCACTCACTCACCGACATAAC
	Toll pathway	Reverse CAAACTAGGCGCCGAATCATA
AAEL007768	MYD (MyD88)	Forward GGAGCTTCCTGCAAACCTAA
	Toll pathway	Reverse TATGGCATCTTCCAGCTTGTC
AAEL007624	REL2	Forward AGCTACCGGCATGAGTTATTC
	IMD pathway	Reverse GCGATACAGATTCCATCGAGAG
AAEL001929	Spatzle5	Forward ACCTCCGGTGAATCACAATC
	Toll pathway	Reverse CAACCATTCCGCTGGACTAA
AAEL027860	Caspar	Forward TCTGAGAATCGCGAGGAGAT
	IMD pathway	Reverse GCGGACAGTAGATCCCAATTAC

**Table 2:** Mayaro virus (MAYV) infection and viral titer in Brazilian and Florida populations of *Aedes aegypti*. Viral titers are expressed in log_10_ plaque forming unit equivalents/mL. Mosquitoes were tested for infection on the day they died following ingestion of MAYV-infected blood.

Treatment	Mosquito Strain	No. Tested	No. infected (%)	Body Titer ^†^ (Non-Disseminated Infection)	No. Disseminated Infection (%)	Body Titer ^†^ (Disseminated Infection)	Leg Titer ^†^
Unexposed (control)	Brazil	57	0 (0)	.	.	.	.
Unexposed (control)	USA (Florida)	74	0 (0)	.	.	.	.
MAYV exposed	Brazil	157	86 (54.78) a	2.78 a	59 (68.60) a	4.30 a	2.52 a
MAYV exposed	USA (Florida)	106	34 (32.08) b	2.58 a	14 (41.18) b	3.98 a	3.02 a

**^†^** Treatment groups with the same letter in the same column are not significantly different by comparisons of means.

**Table 3:** PROC LIFETEST results of geographic origin, infection status, and interaction on adult survival of populations of *Aedes aegypti* from Brazil and Florida following oral exposure to Mayaro virus.

Source	*d.f.*	*χ* ^2^	*P*
Geographic origin	1	7.55	0.0060
Infection status	2	2.06	0.3566
Origin × infection status	5	11.28	0.0461

*d.f.*, degrees of freedom.

**Table 4:** Analysis of variance results of geographic origin, infection status, and interaction on the number of eggs laid by populations of *Aedes aegypti* from Brazil and Florida following oral exposure to Mayaro virus.

Source	*d.f.*	*F*	*P*
Geographic origin	1	0.16	0.6870
Infection status	2	6.39	0.0019
Origin × infection status	2	0.10	0.9070
Error	387		

*d.f.*, degrees of freedom.

**Table 5:** Gene expression of antiviral immune related genes following ingestion of Mayaro virus infectious blood in *Aedes aegypti.*

		Brazil (Control)		Brazil (Mayaro)		Florida (Control)		Florida (Mayaro)	
Gene	Time (h)	Mean	Stdev	Mean	Stdev	Mean	Stdev	Mean	Stdev
**caspar**	0 h	1.007	0.138	1.001	0.054	1.019	0.237	1.003	0.088
	4 h	1.002	0.077	1.293	0.847	1.049	0.414	1.006	0.128
	8 h	1.006	0.135	1.015	0.206	1.001	0.041	1.008	0.160
	12 h	1.008	0.158	1.008	0.160	1.022	0.248	1.154	0.624
	24 h	1.001	0.044	1.095	0.584	1.005	0.127	1.001	0.064
	72 h	1.001	0.041	1.003	0.100	1.002	0.071	1.003	0.101
	168 h	2.716	2.301	1.853	1.974	0.626	0.834	0.765	1.245
**spz5**	0 h	1.012	0.191	1.137	0.615	1.005	0.129	1.007	0.144
	4 h	1.002	0.078	1.034	0.303	1.018	0.225	1.012	0.185
	8 h	1.083	0.543	1.203	0.753	1.001	0.045	1.018	0.234
	12 h	1.109	0.596	1.103	0.608	1.001	0.054	1.002	0.081
	24 h	1.111	0.533	1.237	0.848	1.005	0.120	1.044	0.390
	72 h	1.110	0.557	1.072	0.510	1.056	0.447	1.128	0.663
	168 h	2.326	2.209	0.250	0.108	1.406	1.192	0.209	0.204
**myd88**	0 h	1.015	0.213	1.002	0.069	1.014	0.205	1.008	0.154
	4 h	1.030	0.296	1.002	0.079	1.012	0.191	1.007	0.139
	8 h	1.012	0.192	1.073	0.519	1.014	0.213	1.109	0.634
	12 h	1.013	0.200	1.002	0.069	1.012	0.193	1.026	0.280
	24 h	1.020	0.237	1.167	0.834	1.002	0.077	1.002	0.076
	72 h	1.061	0.402	1.010	0.171	1.003	0.087	1.064	0.433
	168 h	0.322	0.261	0.383	0.187	1.533	1.341	1.319	1.171
**rel2**	0 h	1.031	0.312	1.012	0.191	1.004	0.106	1.004	0.105
	4 h	1.002	0.074	1.003	0.092	1.062	0.467	1.007	0.147
	8 h	1.022	0.268	1.016	0.218	1.003	0.093	1.022	0.262
	12 h	1.019	0.248	1.000	0.023	1.007	0.148	1.007	0.150
	24 h	1.102	0.546	1.260	1.089	1.010	0.171	1.022	0.263
	72 h	1.026	0.292	1.006	0.129	1.009	0.167	1.025	0.270
	168 h	2.521	3.224	2.269	1.944	1.693	1.322	3.964	3.422
**rel1**	0 h	1.161	0.685	1.031	0.323	1.094	0.592	1.129	0.586
	4 h	1.064	0.479	1.042	0.338	1.140	0.722	1.101	0.510
	8 h	1.060	0.397	1.289	1.086	1.098	0.613	1.245	0.850
	12 h	1.010	0.176	1.054	0.441	1.061	0.469	1.332	1.270
	24 h	1.280	0.974	1.104	0.516	1.500	1.648	1.878	2.381
	72 h	1.083	0.511	1.403	1.112	1.016	0.218	1.115	0.582
	168 h	1.354	1.028	0.433	0.193	1.960	2.551	0.388	0.391

**Table 6:** Mayaro virus titer (plaque forming unit equivalents/mL) in cohorts of mosquitoes sampled for genes involved in mosquito immune pathways to *Alphaviruses*. For each time point and group, a sample included a pool of 10 mosquitoes, each with three-fold replication.

	Brazil		Florida	
Time (h)	Mean	stdev	Mean	stdev
0 h	4.49	4.14	4.54	4.14
4 h	4.41	4.08	4.51	4.29
8 h	4.36	3.95	4.34	4.04
12 h	4.07	3.65	4.50	4.09
24 h	3.90	3.62	3.82	3.48
72 h	4.98	4.94	2.90	2.74
168 h	3.49	3.54	1.84	1.92
